# Effect of Aerobic Exercise on Inflammatory Markers in Healthy Middle-Aged and Older Adults: A Systematic Review and Meta-Analysis of Randomized Controlled Trials

**DOI:** 10.3389/fnagi.2019.00098

**Published:** 2019-04-26

**Authors:** Guohua Zheng, Pingting Qiu, Rui Xia, Huiying Lin, Bingzhao Ye, Jing Tao, Lidian Chen

**Affiliations:** ^1^College of Nursing and Health Management, Shanghai University of Medicine & Health Sciences, Shanghai, China; ^2^College of Rehabilitation Medicine, Fujian University of Traditional Chinese Medicine, Shangjie University Town, Fuzhou, China; ^3^Fujian Key Laboratory of Rehabilitation Technology, Fujian University of Traditional Chinese Medicine, Shangjie University Town, Fuzhou, China

**Keywords:** aerobic exercise, inflammatory markers, CRP, TNF-α, IL-6, IL-4

## Abstract

**Background:** Chronic inflammation plays a significant role in accelerating the aging process and is closely associated with the initiation and progression of a broad range of age-related diseases. Physical exercise is considered beneficial in alleviating these conditions, but the effects of aerobic exercise on inflammatory markers in a healthy population should be furtherly clarified.

**Objective:** The purpose of this systematic review and meta-analysis was to evaluate the effect of aerobic exercise on inflammatory markers in middle-aged and older adults.

**Methods:** The literature search was conducted utilizing PubMed, Web of Science, Embase, and the Cochrane Library from their inception through April 2018, and the reference lists were screened to identify appropriate studies. Only randomized controlled trials that investigated the effect of aerobic exercise on inflammatory markers in middle-aged and older adults were eligible for this review.

**Results:** Eleven studies involving 1,250 participants were retrieved from the databases for analysis. The pooled results showed that aerobic exercise significantly reduced inflammatory markers (C-reactive protein (CRP): SMD = 0.53, 95% CI 0.26–0.11, *p* = 0.0002; tumor necrosis factor-alpha (TNF-α): SMD = 0.75, 95% CI 0.31–1.19, *p* = 0.0007; interleukin 6 (IL-6): SMD = 0.75, 95% CI 0.31–1.19, *p* = 0.0007). No significant improvement was found in relation to interleukin 4 (IL-4).

**Conclusions:** Aerobic exercise may have a positive effect on reduction of CRP, TNF-α, and IL-6 in middle-aged and older adults. Further randomized controlled trials (RCTs) need to be conducted to determine the effect of aerobic exercise on additional inflammatory markers in the population of middle-aged and older adults.

## Introduction

Aging is a complex process that is compounded by a combination of environmental, genetic, and epigenetic factors. Chronic inflammation plays an increasingly significant role in health status by accelerating the aging process (Franceschi and Campisi, 2014). Low-grade, persistent chronic inflammation occurs in the majority of the middle-aged and elderly population and is thought to be an accelerator of biological aging (Fougère et al., 2016). An increasing number of studies have demonstrated that chronic inflammation is closely associated with the initiation and progression of a broad range of age-related diseases, such as cardiovascular disease, cancer, diabetes, Alzheimer's disease, and other neurodegenerative diseases and is an independent risk factor for mortality in healthy adults (Kalogeropoulos et al., 2010; Argilés et al., 2015; Bonaccio et al., 2016; Landman et al., 2016; Uchoa et al., 2016; Korniluk et al., 2017; Lang et al., 2018). Moreover, there is strong evidence that the development of age-related diseases is linked to low-grade elevation of circulating inflammatory mediators (Singh and Newman, 2011). Therefore, future interventional researches should focus on preserving overall homeostatic balance and controlling inflammatory status in the aging patient.

Physical exercise is well-recognized as an important strategy for reducing the risk of age-associated diseases (Sparling et al., 2015), and recent research has focused on the role of exercise in the improvement of the inflammatory profile. Large population-based cross-sectional and cohort studies consistently show an inverse association between markers of systemic inflammation and physical exercise; lower inflammatory biomarker concentration is observed in subjects with more frequent and more intense physical exercise (Colbert et al., 2004; Beavers et al., 2010; Lee et al., 2012; Streese et al., 2018). However, data from interventional studies designed to definitively examine the effects of physical exercise on inflammation are limited, and results are inconclusive. For example, in Woods' review of randomized clinical trials, there was evidence indicating that regular exercise could induce loss of fat mass and adipose tissue, which is known to contribute to systemic inflammation (Woods et al., 2012). Another meta-analysis of eight RCTs also showed that exercise could reduce inflammatory markers in older adults, thereby decreasing the risk of developing age-related diseases (Monteiro Junior et al., 2017). Several meta-analyses or systematic reviews indicated that exercise had a beneficial effect in reducing inflammation in patients with chronic diseases, such as breast cancer (Meneses-Echavez et al., 2016), heart disease (Hammonds et al., 2016), chronic cord injury (Neefkes-Zonneveld et al., 2015), and diabetes (Hayashino et al., 2014), but there is no consensus regarding the effect of regular practice of exercise on the circulating inflammatory biomarkers in the relatively healthy adults. In a study by Nicklas et al. the results did not find that exercise training had a significant effect on the inflammatory biomarkers (including C-reactive protein, interleukin 6, and tumor necrosis factor alpha receptor 1) in community-dwelling, older, overweight or obese sedentary adults (Nicklas et al., 2004); others also failed to present positive effects (Walker, 2010; Sahl et al., 2017). Possible reasons for this discrepancy are likely related to differences in exercise type (e.g., aerobic vs. resistance), differences between study participants (e.g., age, sex, health status, and baseline inflammation), differences in exercise protocols (e.g., intensity, frequency, and duration of intervention), or the publication of underpowered findings. Furthermore, current systematic reviews and meta-analyses have not investigated the effects of different exercise types on inflammatory markers in healthy adults, particularly middle-aged and elderly adults. A recent systematic review revealed inconsistent findings related to the effect of aerobic and resistance training on the inflammatory markers CRP and IL-6 (Cronin et al., 2017). Therefore, the present study sought to critically evaluate the effects of aerobic exercise on inflammatory makers of healthy middle-aged and elderly adults through a systematic review and meta-analysis of randomized controlled trials.

## Methods

### Search Strategy

To identify eligible studies, a literature search was conducted in the electronic databases PubMed, Web of Science, the Cochrane Library, and Embase from database inception through April 4, 2018 using combinations of Medical Subject Headings (MeSH) or free text words and the concepts of aerobic exercise training, inflammatory markers, and age; the search was free of restriction to region or publication type. The reference lists of retrieved studies were also screened to identify additional relevant articles. A complete search strategy is provided in the Supplementary Material.

### Inclusion Criteria

Studies were included in the review only if the following criteria were met: (1) Study type: Randomized controlled trials (RCTs); (2) Participants: Middle-aged and older adults (40 years and older) without a disease or medical condition; (3) Intervention: Any style of aerobic exercise or aerobic exercise combined with non-exercise interventions were performed by the experimental group for at least 4 weeks, with three or more sessions every week; (4) Control: no exercise intervention was performed expect for usual level of activity and the sham exercise (e.g., stretch, balance); (5) Outcomes: One or more inflammatory markers were measured in serum or plasma. Studies not written in English or that were without available data were excluded.

### Study Selection and Data Extraction

During the preliminary screening, all searched records were imported into reference management software (NoteExpress V 3.2.0) to eliminate duplicate records and identify potential eligibility by screening titles and abstracts. Then, full-text review was performed. All discrepancies were resolved by a reviewer (GHZ). Data were extracted by one reviewer (PTQ), using a predefined form, and verified by another reviewer (GHZ). The data included the first author; study characteristics (e.g., year, design, and methodological information); participant characteristics (e.g., mean age, sample size); intervention for the experimental and control group (e.g., duration, frequency, intensity, and style of aerobic exercise); outcome findings.

### Assessment of Risk of Bias of Included Studies

The risk of bias of included studies was assessed by two independent reviewers (PTQ and RX) using the Cochrane Collaboration tool (Higgins and Green, 2011). The tool includes seven key items divided into 6 domains: (1) selective bias (random sequence generation and allocation concealment), (2) performance bias (blinding of participants and personnel), (3) detection bias (blinding of outcome assessors), (4) attrition bias (incomplete outcome data), (5) reporting bias (selective reporting), and (6) other bias. For each study, each individual item was assessed, and each domain was graded as “low,” “high” or “unclear,” based on whether the domain met the evaluation criteria with respect to the characteristic expressed by the items. A third reviewer (GHZ) was invited to resolve any disparities.

### Data Analysis

Statistical analyses were conducted using Review Manager 5.3 software (RevMan 5.3). We conducted a meta-analysis to determine change in inflammatory markers from baseline to post-intervention by calculating the standardized mean difference (SMD) between the experimental and control groups, with a 95% confidence interval (CI). The SMD and the standard error (SE) of each inflammatory marker, both before and after treatment, were calculated utilizing Morris's formula (Morris, 2008). If the data were reported as mean and 95% CI, SD was calculated using RevMan software. If the data were reported as median interquartile range (IQR), we calculated mean and standard deviation utilizing the Wan and Luo formulae (Wan et al., 2014; Luo et al., 2015). If a study reported only the value change of the inflammatory markers, we contacted the author to obtain the original data. Data were pooled for meta-analysis when two or more studies measured the same outcome and provided data in a format suitable for pooling. The heterogeneity among the included studies was assessed using a χ^2^ test and Higgins *I*^2^ value. With the χ^2^ test, *P* < 0.05 was considered to be significant. The pooled effect was calculated using the fixed-effect model when data were available, and there was no significant heterogeneity detected. Otherwise, the random-effect model was applied.

## Results

### Study Selection

**Figure 1:**
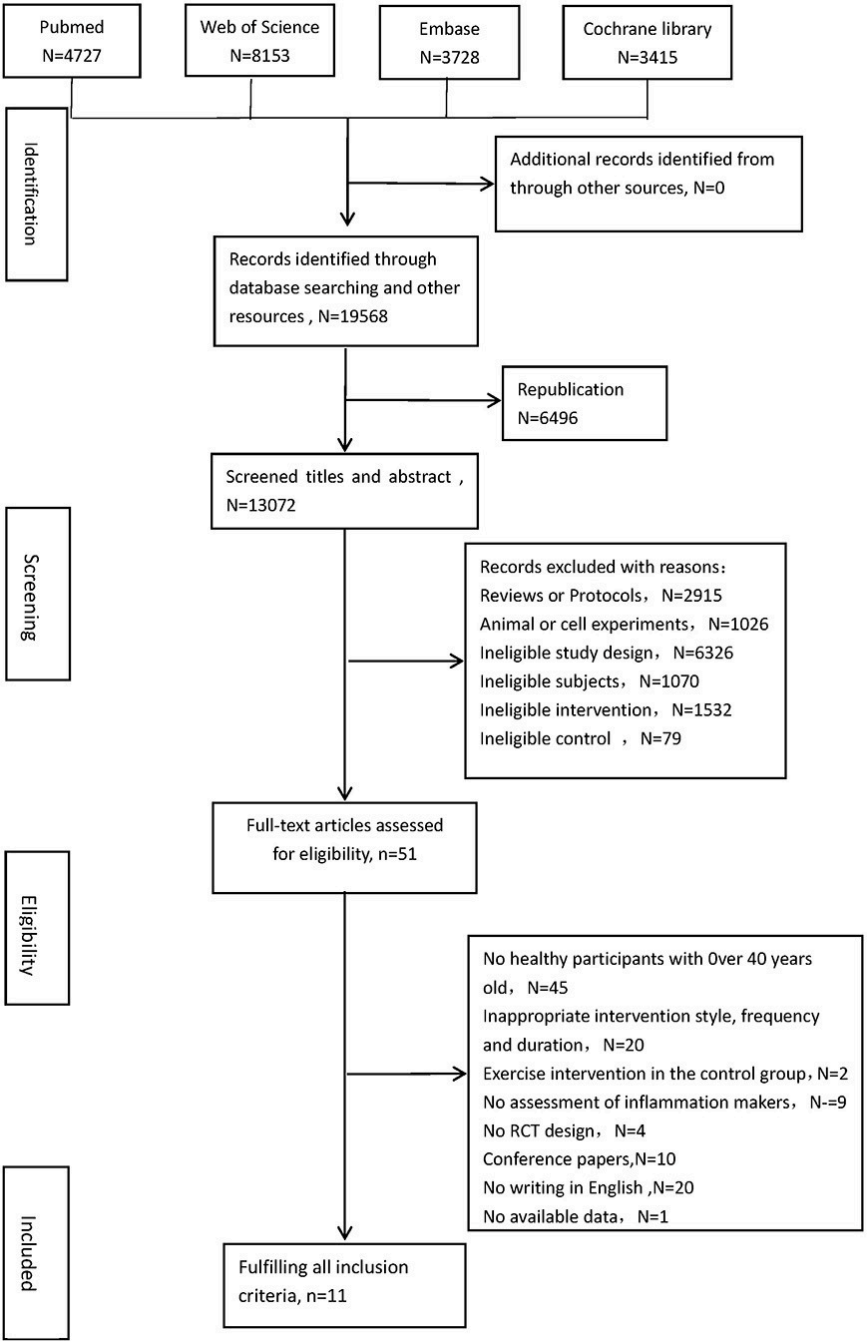
Flow diagram for search and selection of the included studies.

Utilizing the search strategy, 19,568 records were identified from the four electronic databases. After deleting duplicates, two reviewers (PTQ and RX) screened titles and abstracts and excluded unrelated records. Finally, 122 full-text articles were examined for eligibility, and 12 studies met the inclusion criteria (Bergström et al., 2009; Muscari et al., 2010; Tartibian et al., 2011, 2015; Friedenreich et al., 2012; Irwin and Olmstead, 2012; Nishida et al., 2015; Abdollahpour et al., 2016; Alghadir et al., 2016; Conroy et al., 2016; Sbardelotto et al., 2017; Mohammadi et al., 2018). One of these 12 studies (Sbardelotto et al., 2017) was excluded, as data remained unavailable, despite attempts to contact the original author. Thus, 11 studies were included in the review. The study selection flowchart for locating eligible articles is provided in Figure 1.

### Characteristics of Included Studies

The characteristics of the included articles are presented in Table 1. A total of 11 RCTs involving 1,250 participants (188 males and 1,062 females, age range 40–95 years) were included in the review. The styles of aerobic exercise utilized in the intervention groups were diverse, involving Tai Chi (Irwin and Olmstead, 2012), treadmill (Mohammadi et al., 2018), bench step exercises (Nishida et al., 2015), and multicomponent aerobic exercises (Bergström et al., 2009; Muscari et al., 2010; Tartibian et al., 2011, 2015; Alghadir et al., 2016); three studies (Friedenreich et al., 2012; Abdollahpour et al., 2016; Conroy et al., 2016) did not describe a specific exercise style. The duration of the exercise phase in the included studies ranged from 2 to 12 months. The frequency of exercise varied from two to five sessions weekly, with each session lasting 20–90 min. Of the 11 studies, 8 assessed exercise intensity through the heart rate reserve of 45–80% of maximum heart rate (Muscari et al., 2010; Tartibian et al., 2011, 2015; Friedenreich et al., 2012; Abdollahpour et al., 2016; Alghadir et al., 2016; Conroy et al., 2016; Mohammadi et al., 2018). The remaining studies used the following strategies: (1) “at least 95% at training intensity level 3–4 (1–3 walks and 1–2 aerobic exercise/week)” (Bergström et al., 2009); (2) “moderate intensity” (Irwin and Olmstead, 2012); and (3) “an intensity of lactate threshold” (Nishida et al., 2015). All control groups were described as maintaining their usual physical activity.

**Table 1:** Characteristics of included studies in this systematic review.

**References**	**Age (range)**	**Participant(M/F)**	**Intervention**	**Frequency, duration, and intensity of aerobic exercise**	**Findings**
Abdollahpour et al., 2016	50–74 years	41 (0/41)	E:aerobic exercise C:maintain their usual physical activity levels	50 min/day,3 days/week for 6 months; 70–80% HRmax	↔TNF-a,↓IL-6 when E compare to C at post-test
Alghadir et al., 2016	65–95 years	100 (70/30)	E: treadmill, bicycle, and StairMaster C:maintain their usual lifestyle	45–60 min/time,3 times/week for 24 weeks; 60–70% HRmax	↔hsCRP when E compare to C at post-test
Bergström et al., 2009	45–65 years	112 (0/112)	E:brisk walk C:maintain their usual physical activity level	30 min brisk walk + 60 min aerobic exercise/day, 3 brisk walks, and 1–2 aerobic exercises/week for 24 week; at least 95% level 3–4 training intensity (Level3:1–3 walks +1 aerobic session/week; Level 4: 3 walks +2 aerobic session/week).	↔hsCRP when E compared to C at post-test
Conroy et al., 2016	50–74 years	320 (0/320)	E:aerobic exercise C: maintain their usual level of activity	45 min/session, 5 session/week, for 1 year; 70–80% HRmax	↔ IL-4, ↔ IL-10 when E compare to C at post-test
Friedenreich et al., 2012	50–74 years	210 (0/210)	E:aerobic exercise C:usual inactivity	45 min/day, 5 days/week for 1 year; 70–80% HRmax	↓hsCRP,↔TNF-α, ↔IL-6 when E compare to C at post-test
Irwin and Olmstead, 2012	59–86 years	83 (32/51)	E: Tai Chi Chih C:health education	40 min/session,3 session/week for 16 weeks; Moderate intensity	↓IL-6, ↔ hsCRP, ↔IL-18 when E compare to C at post-test
Mohammadi et al., 2018	40–60 years	24 (24/0)	E: treadmill C:usual lifestyle	20–60 min/session,3 times/week for 12 weeks; 60–70% HRmax	↔hsCRP,↔ICAM-1,↔VCAM-1 when E compare to C at post-test
Muscari et al., 2010	65–74 years	120 (62/58)	E:Cycle ergometer, treadmill and free-body activity C:health education	60 min/session, 3 times/week for 1 year; 70% HRmax	↓hsCRP when E compare to C at post-test
Nishida et al., 2015	65–85years	62 (0/62)	E:bench step exercise C:maintain their usual lifestyle	10–20 min/session,3 times/day, and a goal of 140 min/week for 12 weeks; lactate threshold	↔ IL−4,↔ IL-5,↔ IL-6, ↔ IL-8, ↔ IL-15, ↔ TNF-α, ↔ TNF-β, ↓IFN-γ when E compare to C at post-test
Tartibian et al., 2011	58–78 years	38 (0/38)	E: walking or jogging C:maintain their usual physical activity levels	25–30 min/day,3–4days/week with 45–55% HRmax for the first 12 weeks; then 40–45 min/day,4–6 days/week with 55–65% HR for the second 12 weeks	↓TNF-a, ↓IL-6, ↓PGE_2_ when E compare to C at post-test
Tartibian et al., 2015	50–65 years	28 (0/28)	E:walking or jogging C:maintain their usual physical activity levels	25–30 min/day,3–4 days/week with 45–55% HRmax for the first 8 weeks; 40–45 min/day,4–6 days/week with 55–65% HR for the final 8 weeks	↓ IL-1β, ↓IL-6, ↓TNF-a, ↓hsCRP when E compare to C at post-test

*E, exercise group; C, control group; ↓, significant reduction; ↔, significant increase; ↔, no change; CRP, C-reactive protein; TNF-α, tumor necrosis factor-alpha; IL-6, interleukin 6; IL-4, interleukin 4; INF-γ, interferon-gamma; PGE_2_, prostaglandin E_2_; IL-1β, interleukin-1beta; IL-5, interleukin-5; IL-8, interleukin-8; IL-10, interleukin-10; IL-15, interleukin-15; IL-18, interleukin-18; ICAM-1, intercellular adhesion molecule-1; VCAM-1, vascular cell adhesion molecule-1; TNF-β tumor necrosis factor-beta*.

### Risk of Bias of the Included Studies

**Figure 2:**
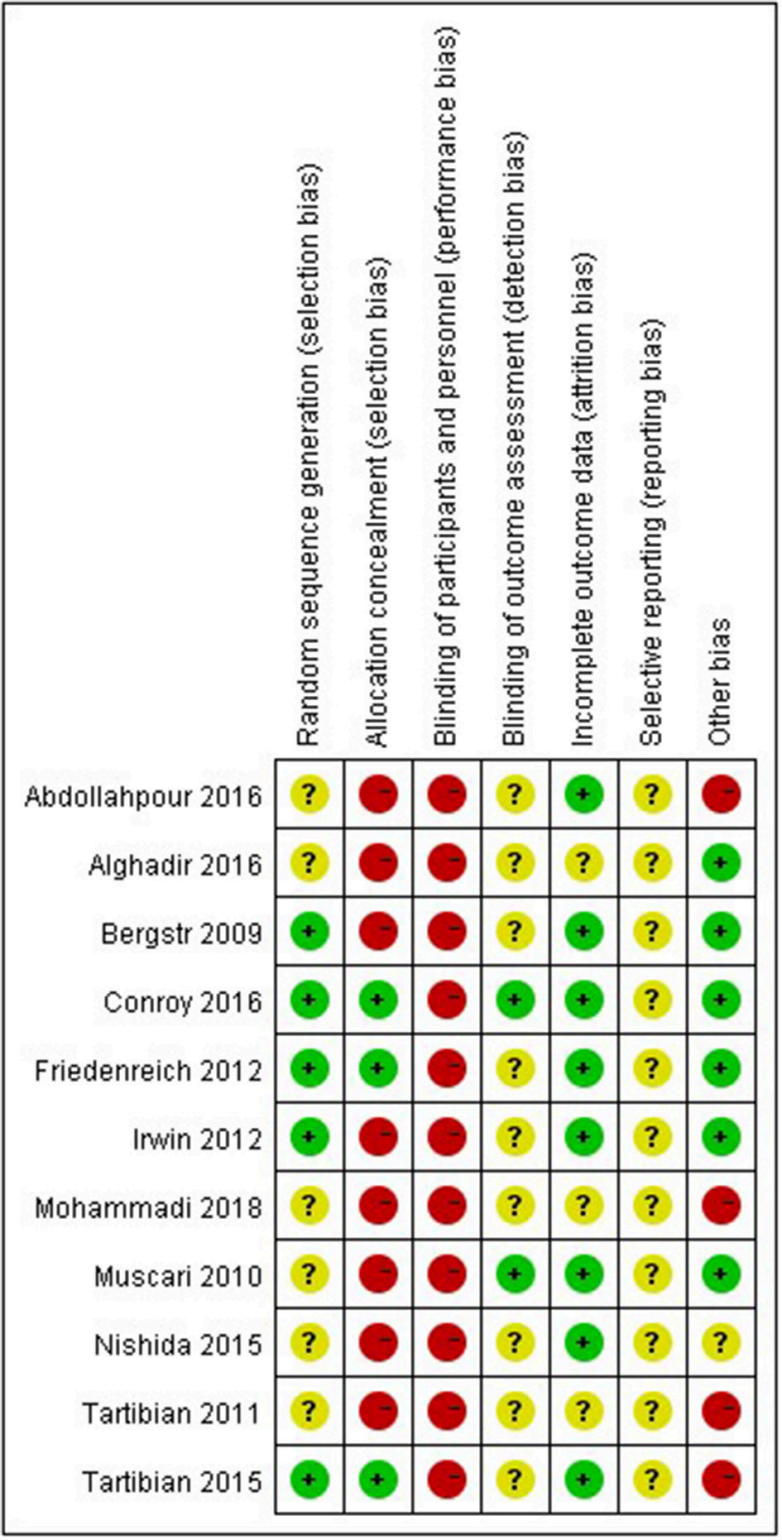
Risk of bias in included studies: the authors' judgments on each risk of bias item for all included studies.

The risk of bias of the included studies is displayed in Figure 2. All of the included articles reported randomized group allocation, but only five trials (Bergström et al., 2009; Friedenreich et al., 2012; Irwin and Olmstead, 2012; Tartibian et al., 2015; Conroy et al., 2016) described the specific method of randomization, and three of those studies (Friedenreich et al., 2012; Tartibian et al., 2015; Conroy et al., 2016) reported allocation concealment. All of the included studies demonstrated high performance bias, as participants and personnel were not blind to the exercise intervention. Two studies (Muscari et al., 2010; Conroy et al., 2016) clearly described blind assessment of outcome measures. Intention-to-treat analysis was used in five studies (Bergström et al., 2009; Muscari et al., 2010; Friedenreich et al., 2012; Abdollahpour et al., 2016; Conroy et al., 2016), and three studies (Irwin and Olmstead, 2012; Nishida et al., 2015; Tartibian et al., 2015) described reasons why participants dropped out or failed to follow up. The risk of other bias in four studies was judged as “high” due to limited sample size (Tartibian et al., 2011, 2015), disproportionate dropout rates between the groups (Abdollahpour et al., 2016), and lack of baseline measurements (Mohammadi et al., 2018).

### Effect of Interventions

Pre-intervention and post-intervention data were reported using mean with standard deviation (SD), or mean with 95% confidence interval, in 10 studies (Muscari et al., 2010; Tartibian et al., 2011, 2015; Friedenreich et al., 2012; Irwin and Olmstead, 2012; Nishida et al., 2015; Abdollahpour et al., 2016; Alghadir et al., 2016; Conroy et al., 2016; Mohammadi et al., 2018), and median (IQR) or median (range) in one study (Bergström et al., 2009). Of the 15 inflammatory markers measured in the included studies, only CRP, TNF-α, IL-6, and IL-4 were reported in more than two trials, rendering the data suitable for meta-analysis.

**Figure 3:**
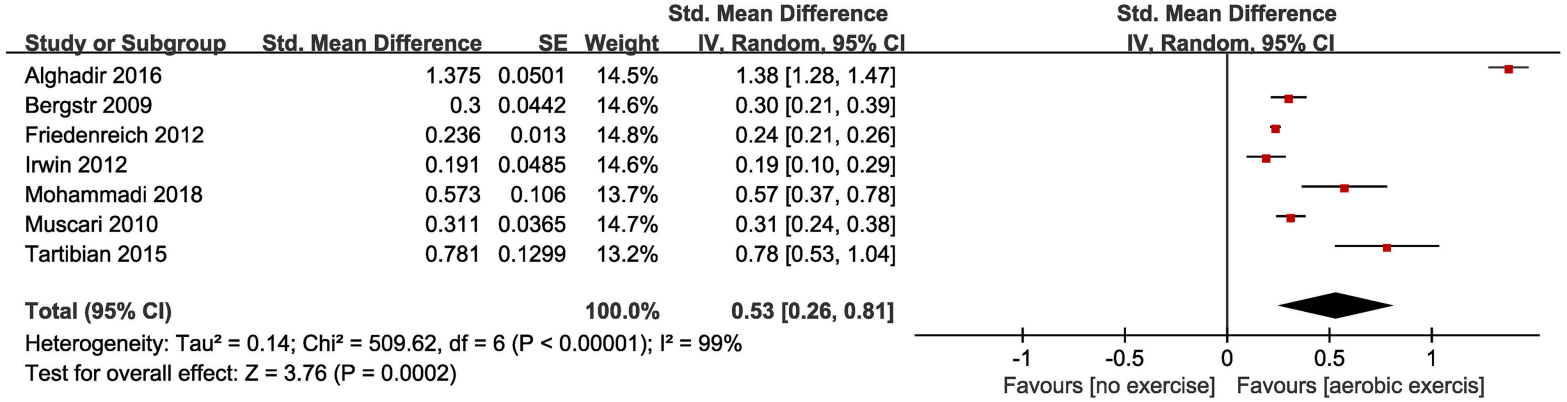
Forest plots of the effect of aerobic exercise on CRP compared with no exercise.

The effect of aerobic exercise on levels of CRP was measured in seven studies. Seven studies (Bergström et al., 2009; Muscari et al., 2010; Friedenreich et al., 2012; Irwin and Olmstead, 2012; Tartibian et al., 2015; Alghadir et al., 2016; Mohammadi et al., 2018) with 774 participants reported appropriate data. Three studies (Muscari et al., 2010; Friedenreich et al., 2012; Tartibian et al., 2015) demonstrated a significantly lower post-intervention level of CRP in the exercise group compared to the control group. The pooled SMD showed a statistically significant decrease in CRP (SMD = 0.53, 95% CI 0.26–0.81, *P* = 0.0002; Figure 3).

**Figure 4:**
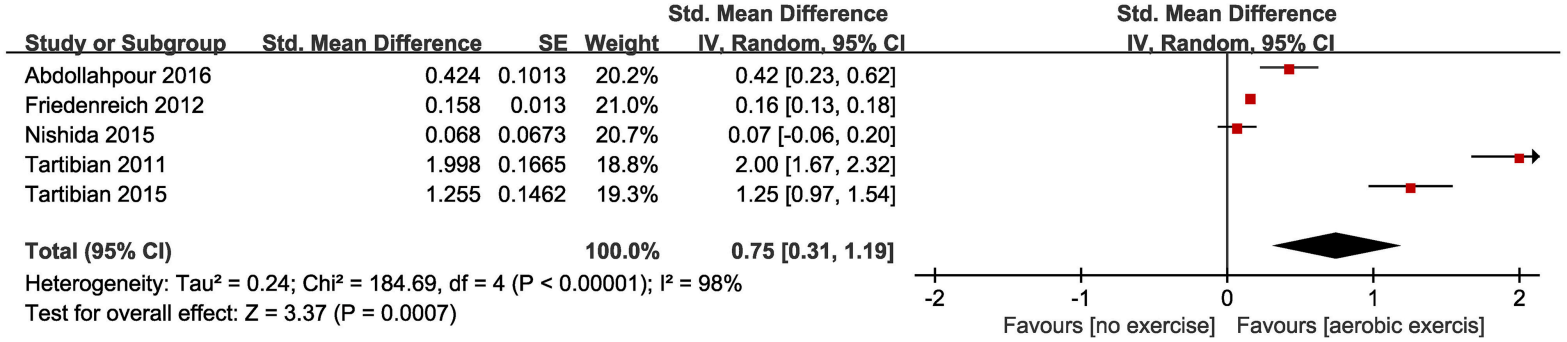
Forest plots of the effect of aerobic exercise on TNF-α compared with no exercise.

Five of the included studies (Tartibian et al., 2011, 2015; Friedenreich et al., 2012; Nishida et al., 2015; Abdollahpour et al., 2016) looked at TNF-α levels, and two of these five(Tartibian et al., 2011, 2015) found a significant post-intervention reduction of the level of TNF-α in the exercise group compared with the control group; however, when the data from these five studies were pooled, SMD between the studies demonstrated a significant reduction (SMD = 0.75, 95% CI 0.31–1.19, *P* = 0.0007; Figure 4).

**Figure 5:**
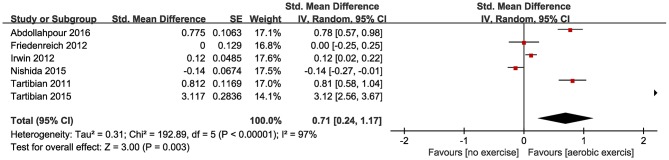
Forest plots of the effect of aerobic exercise on IL-6 compared with no exercise.

Six of the included studies investigated IL-6 (Tartibian et al., 2011, 2015; Friedenreich et al., 2012; Irwin and Olmstead, 2012; Nishida et al., 2015; Abdollahpour et al., 2016), and four (Tartibian et al., 2011, 2015; Irwin and Olmstead, 2012; Abdollahpour et al., 2016) of these studies identified a significant reduction in IL-6 levels for the aerobic exercise group compared to the control group. The forest plots also showed aerobic exercise significantly reduced IL-6 levels in healthy middle-aged and older adults (SMD = 0.71, 95% CI 0.24–1.17, *P* = 0.003; Figure 5).

**Figure 6:**
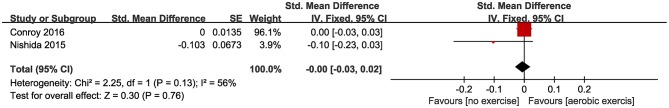
Forest plots of the effect of aerobic exercise on IL-4 compared with no exercise.

Only two of the eligible studies (Nishida et al., 2015; Conroy et al., 2016) reported IL-4 levels, and neither reported a statistically significant decrease in IL-4 levels for the aerobic exercise group compared with the control group. Likewise, the pooled SMD did not show significant changes between the aerobic exercise and control groups (SMD = 0.00, 95% CI −0.03 to 0.02, *P* = 0.76; Figure 6).

Three studies (Tartibian et al., 2011, 2015; Nishida et al., 2015) that measured the concentration of interferon-gamma (INF-γ), prostaglandin E_2_ (PGE_2_), and interleukin-1beta (IL-1β), respectively, demonstrated a statistically significant reduction in the levels of these markers for the aerobic exercise group compared to controls; other studies (Irwin and Olmstead, 2012; Nishida et al., 2015; Conroy et al., 2016; Mohammadi et al., 2018), involved individually in assessing interleukin-5 (IL-5), interleukin-8 (IL-8), interleukin-10 (IL-10, interleukin-15 (IL-15), interleukin-18 (IL-18), intercellular adhesion molecule-1 (ICAM-1), vascular cell adhesion molecule-1 (VCAM-1), and tumor necrosis factor-beta (TNF-β) levels, failed to find statistically significant differences between the aerobic exercise groups and controls.

### Adverse Events

No adverse events were reported in the studies included in this review.

## Discussion

This systematic review and meta-analysis sought to evaluate the relationship between aerobic exercise and inflammatory markers in healthy middle-aged and older adults. Eleven randomized controlled trials, with a total of 1,138 participants, that compared aerobic exercise groups with no-exercise control groups were included in this review. The results demonstrate that regular aerobic exercise has a positive effect on decreasing most of the reported inflammatory makers, including CRP, TNF-α, and IL-6, in healthy middle-aged and older adults.

There are strong correlations between inflammation and aging. The prospective epidemiological studies have found an increased risk of age-related chronic diseases is associated with increased basal-level inflammation (Ridker et al., 2000). As a result, aging is associated with low-grade inflammation (Nagano et al., 2005), and increases in circulating levels of inflammatory markers have been observed in the aged populations (Ogawa et al., 2008). It is established that with age, physiological processes generate adaptations within the immune system, resulting in a continuous response of factors that trigger a chronic inflammatory response (Ostan et al., 2008), including increased fat tissue, decreased sex steroid production, and chronic disorders (Woods et al., 2012). Exercise has direct effects on the cellular immune system, as cytotoxic immune cells can be mobilized into circulation through adrenergic signaling during exercise performance (Idorn and Hojman, 2016). Therefore, exercise is recommended as an appropriate non-pharmacological strategy to modulate the systemic inflammatory status; however, many previous reviews have been limited to studying exercise effects on inflammation in individuals with disease (Hayashino et al., 2014; Neefkes-Zonneveld et al., 2015; Hammonds et al., 2016; Meneses-Echavez et al., 2016). A recent systematic review reported regular exercise training may decrease levels of CRP and IL-6, but no statistical significance was found for TNF-α in an elderly population (Monteiro Junior et al., 2017). Another meta-analysis, not limited to a specific clinical population, indicated that exercise training could lower CRP levels in individuals (Fedewa et al., 2017). However, considering the methodological diversity among the included studies, particularly the differences between exercise types (i.e., aerobic, resistance) and different basal levels of cytokines, the results of those meta-analysis or review should be interpreted cautiously. The present systematic review focused on the effect of aerobic exercise on inflammatory markers in healthy middle-aged and older adults. Following the initial screening of over 13,000 article references, 11 randomized controlled trials were ultimately included in the review. The pooled results revealed aerobic exercise led to a significant reduction of inflammatory markers, including CRP, TNF-α, and IL-6. Furthermore, all included studies adhered to a pretest-posttest-control design, in which participants are randomly assigned to the treatment or control group, and each participant is measured both before and after the treatment. Considering that preexisting differences between groups can artificially inflate or obscure posttest differences, we used the Morris method to adjust the heterogeneity of inflammatory markers at baseline (before treatment) among the included studies (Morris, 2008). Therefore, the results in this review were more robust than findings of previous studies.

This study has several limitations. First, significant heterogeneity was found in the CRP, TNF-α, and IL-6 data among the included studies; a possible cause may be that inflammatory marker levels are affected by different methods of collection and sample preparation, as well as by time elapsed between previous exercise session and plasma or serums measurement (Wu et al., 2007). Additionally, there were discrepancies between the included studies regarding different types, duration, and frequencies of the aerobic exercise interventions. These are all potential sources of differences in results between studies. However, due to an insufficient number of studies of each inflammatory marker, sub-analysis could not be performed. Second, the pooled effect for other inflammatory markers, such as IL-8, IL-10, and VCAM-1, could not be analyzed due to an insufficient number of included studies that assessed these markers. Thus, additional randomized controlled trials should be conducted to determine the effects of aerobic exercises on other inflammatory markers. Third, studies not reported in English were excluded, which could have led to publication bias; however, funnel plot analysis to detect publication bias was not possible due to the limited number of included studies (less ten studies for each outcomes). Finally, relating to the specificity of intervention, it is not feasible to blind participants and exercise trainers during aerobic exercise intervention; therefore, performance bias may be inevitable.

## Conclusions

This review reveals that aerobic exercise has significant benefits on levels of CRP, TNF-α, and IL-6. Considering the limited number of included studies, considerably larger-sample size RCTs are necessary to determine the effect of aerobic exercise on additional inflammatory markers in middle-aged and older adults.

## Author Contributions

LC, GZ, and JT conceived and designed the study. PQ, RX, HL, and BY performed the search, extraction of data, and methodological assessment. PQ and GZ analyzed the data and wrote the paper. All authors read and approved the final manuscript.

### Conflict of Interest Statement

The authors declare that the research was conducted in the absence of any commercial or financial relationships that could be construed as a potential conflict of interest.
