# Supplementary material for: Effect of Aerobic Exercise on Inflammatory Markers in Healthy Middle-Aged and Older Adults: A Systematic Review and Meta-Analysis of Randomized Controlled Trials
Source: Front Aging Neurosci. 2019 Apr 26;11:98. doi: 10.3389/fnagi.2019.00098 (PMC6497785; doi:10.3389/fnagi.2019.00098)
Supplement: Supplementary file 1 [file Table_1.docx]

Supplementary Material

Effect of aerobic exercise on inflammatory markers in healthy middle-aged and older adults: A systematic review and meta-analysis of randomized controlled trials

**Guohua Zheng^1,2#^, Pingting Qiu^2#^, Rui Xia^2^, Huiying Lin^2^, Bingzhao Ye^2^, Jing Tao^2^, Lidian Chen^2,3^***

^1^ College of Nursing and Health Management, Shanghai University of Medicine & Health Sciences. Pudong New District, Shanghai, China

^2^ College of Rehabilitation Medicine, Fujian University of Traditional Chinese Medicine, Shangjie University Town, Fuzhou, China

^3^ Fujian Key Laboratory of Rehabilitation Technology, Fujian University of Traditional Chinese Medicine, Shangjie University Town, Fuzhou, China;

*** Correspondence:**

Lidian Chen:lidianchen87@163.com

#Guohua Zheng and Pingting Qiu contributed equally to this work.

**Search strategies**

**Pubmed**

#1 aerobic exercise [ALL]

#2 exercise [ALL]

#3 fast walking [ALL]

#4 tai ji [ALL]

#5 yoga [ALL]

#6 brisk walking [ALL]

#7 jogging [ALL]

#8 taijiquan [ALL]

#9 tai chi [ALL]

#10 qigong [ALL]

#11 physical activity [ALL]

#12 physical exercise [ALL]

#13 physical endurance [ALL]

#14 treadmill [ALL]

#15 bicycling [ALL]

#16 dance [ALL]

#17 rope skipping [ALL]

#18 #1—#17/OR

#19 older [ALL]

#20 elderly [ALL]

#21 age [ALL]

#22 #19—#21/OR

#23 inflamma* [ALL]

#24 cytokines [ALL]

#25 interleukin [ALL]

#26 Tumor Necrosis Factor [ALL]

#27 Transforming growth factor[ALL]

#28 C-Reactive protein [ALL]

#29 IL-1 [ALL]

#30 IL-6 [ALL]

#31 IL-8 [ALL]

#32 TNF-α [ALL]

#33 TGF-β [ALL]

#34 CRP [ALL]

#35 #23—#34/OR

#36 control [ALL]

#37 controlled trial [ALL]

#38 comparison [ALL]

#39 #36—#38/OR

#40 #18 AND #22 AND #35 AND #39

**web of Science**

#1 TS:( aerobic exercise )

#2 TS: (exercise )

#3 TS: (fast walking )

#4 TS: (tai ji )

#5 TS: (yoga )

#6 TS: (brisk walking )

#7 TS: (jogging )

#8 TS: (taijiquan )

#9 TS: (tai chi )

#10 TS:(qigong )

#11 TS:(physical activity )

#12 TS:(physical exercise )

#13 TS:(physical endurance )

#14 TS:(treadmill )

#15 TS:(bicycling )

#16 TS:(dance )

#17 TS:(rope skipping )

#18 #1—#17/OR

#19 TS:( older )

#20 TS:(elderly )

#21 TS:(age )

#22 #19—#21/OR

#23 TS:( inflamma* )

#24 TS:(cytokines)

#25 TS:(interleukin )

#26 TS:(Tumor Necrosis Factor )

#27 TS:(Transforming growth factor)

#28 TS:(C-Reactive protein )

#29 TS:( IL-1 )

#30 TS:( IL-6 )

#31 TS:( IL-8 )

#32 TS:( TNF-α )

#33 TS:(TGF-β)

#34 TS:(CRP )

#35 #23—#34/OR

#36 TS:(control)

#37 TS: (controlled trial )

#38 TS:( comparison)

#39 #36—#38/OR

#40 #18 AND #22 AND #35 AND #39

**Embase**

#1 'aerobic exercise'/exp OR 'aerobic exercise'

#2 'exercise'/exp OR 'exercise'

#3 'fast walking'

#4 'tai ji'

#5 'yoga'/exp OR 'yoga'

#6 'brisk walking'/exp OR 'brisk walking'

#7 'jogging'/exp OR jogging

#8 'taijiquan'/exp OR taijiquan

#9 'tai chi'/exp OR 'tai chi'

#10 'qigong'/exp OR qigong

#11 'physical activity'/exp OR 'physical activity'

#12 'physical exercise'/exp OR 'physical exercise'

#13 'physical endurance'/exp OR 'physical endurance'

#14 'treadmill'/exp OR treadmill

#15 'bicycling'/exp OR bicycling

#16 'dance'/exp OR dance

#17 'rope skipping'

#18 #1-#17/OR

#19 older

#20 elderly

#21 age

#22 #19—#21/OR

#23 inflamma*

#24 'cytokines'/exp OR cytokines

#25 'interleukin'/exp OR interleukin

#26 'tumor necrosis factor'/exp OR 'tumor necrosis factor'

#27 'transforming growth factor'/exp OR 'transforming growth factor'

#28 'C-Reactive protein'/exp OR C-Reactive protein

#29 'il 1'/exp OR 'il 1'

#30 'il 6'/exp OR 'il 6'

#31 'il 8'/exp OR 'il 8'

#32 'tnf α'

#33 'tgf β'

#34 ' crp '

#35 #23—#34/OR

#36 control

#37 controlled trial

#38 comparison

#39 #36—#38/OR

#40 #18 AND #22 AND #35 AND #39

**Chcorane Library**

#1 aerobic exercise

#2 exercise

#3 fast walking

#4 tai ji

#5 yoga

#6 brisk walking

#7 jogging

#8 taijiquan

#9 tai chi

#10 qigong

#11 physical activity

#12 physical exercise

#13 physical endurance

#14 treadmill

#15 bicycling

#16 dance

#17 rope skipping

#18 #1—#17/OR

#19 older

#20 elderly

#21 age

#22 #19—#21/OR

#23 inflamma*

#24 cytokines

#25 interleukin

#26Tumor Necrosis Factor

#27Transforming growth factor

#28 C-Reactive protein

#29 IL-1

#30 IL-6

#31 IL-8

#32 TNF-α

#33 TGF-β

#34 CRP

#35 #23—#34/OR

#36 control

#37 controlled trial

#38 comparison

#39 #36—#38/OR

#40 #18 AND #22 AND #35 AND #39
